# Experimental Investigation of Polypropylene Composite Drawn Fibers with Talc, Wollastonite, Attapulgite and Single-Wall Carbon Nanotubes

**DOI:** 10.3390/polym14020260

**Published:** 2022-01-09

**Authors:** Costas Tsioptsias, Konstantinos Leontiadis, Stavros Messaritakis, Aikaterini Terzaki, Panagiotis Xidas, Kyriakos Mystikos, Evangelos Tzimpilis, Ioannis Tsivintzelis

**Affiliations:** 1 Department of Chemical Engineering, Aristotle University of Thessaloniki, GR-54124 Thessaloniki, Greece; leontiad@cheng.auth.gr (K.L.); tzimpi@auth.gr (E.T.); 2 Plastika Kritis S.A., R Street, Industrial Area of Heraklion, GR-71408 Heraklion, Greece; messaritakis@plastikakritis.com (S.M.); terzaki@plastikakritis.com (A.T.); 3 Thrace Nonwovens & Geosynthetics S.A., Magiko, GR-67100 Xanthi, Greece; pxidas@thraceplastics.gr (P.X.); kmystikos@thraceplastics.gr (K.M.)

**Keywords:** drawn polymer fibers, nanocomposites, carbon nanotubes, talc, wollastonite, attapulgite, antioxidant, compatibilizer

## Abstract

Isotactic polypropylene (PP) composite drawn fibers were prepared using melt extrusion and high-temperature solid-state drawing at a draw ratio of 7. Five different fillers were used as reinforcement agents (microtalc, ultrafine talc, wollastonite, attapulgite and single-wall carbon nanotubes). In all the prepared samples, antioxidant was added, while all samples were prepared with and without using PP grafted with maleic anhydride as compatibilizer. Material characterization was performed by tensile tests, differential scanning calorimetry, thermogravimetric analysis and Fourier transform infrared spectroscopy. Attapulgite composite fibers exhibited poor results in terms of tensile strength and thermal stability. The use of ultrafine talc particles yields better results, in terms of thermal stability and tensile strength, compared to microtalc. Better results were observed using needle-like fillers, such as wollastonite and single-wall carbon nanotubes, since, as was previously observed, high aspect ratio particles tend to align during the drawing process and, thus, contribute to a more symmetrical distribution of stresses. Competitive and synergistic effects were recognized to occur among the additives and fillers, such as the antioxidant effect being enhanced by the addition of the compatibilizer, while the antioxidant itself acts as a compatibilizing agent.

## 1. Introduction

Polypropylene (PP) is one of the most common thermoplastics with attractive properties, such as low density, high thermal stability, chemical resistance and capability of being processed with various methods. A large portion of the worldwide produced PP is used in drawn forms, e.g., biaxially drawn films and uniaxially drawn fibers. In general, by drawing, various attractive PP properties arise, such as improvement of tensile strength, reduction in its inherent fragility at low temperatures, and capability of producing heat-shrinkable films [1]. Such improvement of mechanical properties is related to chain alignment/orientation in the direction of drawing.

Depending on drawing parameters, e.g., draw ratio and temperature, the increase in tensile strength and elastic modulus of PP fibers may be significant, i.e., from 2 to 10 times higher [2,3]. In this direction, the drawing of PP fibers has been extensively studied in terms of fiber production and drawing methods, including the use of melt spinning/drawing [4,5,6], ecologically friendly isothermal bath [7], laser-assisted drawing [8,9], temperature gradient multiple stage drawing [10,11], and overdrawing [12]. However, the drawing of polymer matrices and the subsequent improvement of properties present limitations, such as the difficultly in processing low molecular weight polymers and the shrinkage of drawn materials with aging.

A common approach for improving mechanical and thermal properties of polymeric materials is the development of polymer composite structures through the addition of various, mainly inorganic, particles, as reinforcement agents, in the polymer matrix. Provided that a uniform dispersion of particles is achieved and that (thermodynamically) favorable interactions between macromolecules and particles’ surface are present, significant improvement of polymer properties can be observed, e.g., stresses are shared between polymer and particle interface by various mechanisms [13]. It is well known that, besides additive content and nature, the particle size also has a considerable impact on the alteration of polymer properties, with nanostructures prevailing over microstructures [14], since nanoparticles provide higher contact surface area. Thermodynamically favorable polymer–additive interactions are commonly enhanced by the use of compatibilizers and/or appropriate modifications. Chemical modifications can be applied to the additive, to the polymer, or to both of them, in order to obtain optimum results [15].

In the case of PP, which as a polyolefin presents a non-polar and hydrophobic nature, intermolecular interactions with most inorganic additives are rather poor. Nevertheless, the grafting of PP with polar groups, such as maleic anhydride, results in more thermodynamically favored interactions with hydrophilic additives, such as montmorillonite (a phyllosilicate clay), which in turn may result in intercalated and/or exfoliated structures causing a significant improvement of composite properties [16]. Besides montmorillonite, several other reinforcement agents have been used, consisting of three main categories, i.e., mineral clays, carbonaceous fillers and other inorganic nanoparticles, which were recently reviewed [15]. Briefly, mineral clays that were used in PP composites include talc [17], boehmite [18], hectorite [19] and sepiolite, which present a needle-like morphology [20]. Wollastonite has been used for the reinforcement of PP alone with or without modification [21,22], or in combination with other additives, such as silicon rubber [23] and glass fibers [24]. Commonly, wollastonite is used at high loads (up to 40 wt.%). This is the case also for talc. Attapulgite has a nucleating effect for PP [25] and has been used as additive for composite PP materials [26,27].

The studies mentioned above refer to non-drawn PP composites. However, PP drawn fibers constitute a separate category. Since the mechanical properties of PP are significantly enhanced with drawing, further increase by the use of fillers in nanocomposite drawn fibers is a rather difficult task and less studied compared to PP non-drawn composites. In many cases, the deterioration of mechanical properties of PP fibers has been observed after the addition of fillers [15]. Probably, the poor interaction between PP and filler and the inevitable poor dispersion of particles disturbs chain alignment (that governs the increase in mechanical strength) during drawing. For example, the addition of functionalized sepiolite did not improve and in some cases decreased the tenacity of PP yarns [20]. Furthermore, the use of modified hydrotalcite (perkalite) and two differently functionalized montmorillonites in PP drawn fibers resulted in no alteration of tensile at break (for one of the montmorillonites) and in a considerable decrease from 176 MPa to 123 and 136 MPa for the other montmorillonite and the hydrotalcite, respectively [28].

However, in some cases, a considerable improvement was observed. For example, when ZnO particles were used in PP fibers [29], tenacity increased from 31.2 to 39.3 CN/tex. Moreover, very interesting results regarding the reinforcement of PP drawn fibers were observed using nanosilica particles as reinforcement agents [13]. The tensile strength of PP increased from 40.3 to 195.9 MPa by drawing. The addition of 1 wt.% nanosilica did not result in significant change of the tensile strength of the non-drawn sample, but in the drawn sample, the tensile strength increased up to 270.1 MPa. The use of a compatibilizer resulted in a minor increase in the tensile strength regarding the non-drawn sample, but in a major increase regarding the drawn sample (42.9 and 319.6 MPa, respectively). Such results show that despite the tremendous increase in tensile strength with drawing, further (considerable) increase can be achieved by changing the filler, functionalizing the polymer and optimizing filler’s content.

Furthermore, fillers with needle-like structures, which might align in the drawing direction [30], are interesting candidates for the development of composite drawn fibers. In this direction, carbon nanotubes were studied as fillers in PP drawn fibers [20,31]. However, the addition of carbon nanotubes without any functionalization of PP chains did not affect, or deteriorate in some cases, the tensile strength of PP composite drawn fibers, e.g., maximum stress dropped from 30.65 MPa in the neat PP to 18.9 MPa in the composite fibers with 7.5 wt.% single-wall carbon nanotubes [31]. Similarly, oxidation and the introduction of carboxylic groups in carbon nanotubes resulted in decreased tenacity of PP composite yarns with, or without, the use of PP-g-MA as a compatibilizer [20]. On the contrary, proper treatment of fluorinated carbon nanotubes has been reported to cause a considerable improvement of mechanical strength, e.g., from 30.64 to 45.3 MPa using 2.5 wt.% carbon nanotubes, while the corresponding value for 10 wt.% carbon nanotubes was found equal to 77 MPa [31]. The use of various fillers in nanocomposite PP fibers was recently reviewed [15].

However, the use of compatibilizers along with other additives, e.g., antioxidants, coloring substances, lubricants, etc., is a common and essential industrial practice. Interactions of such other additives with compatibilizers and the used fillers are usually not extensively studied, although they may significantly alter the properties of the final polymer matrix. It is likely that the most common compatibilizer for PP is PP-g-MA. In general, additives are mixed with a high melt-flow index (low molecular weight) PP matrix, in order to be more easily dispersed. The lower molecular weight, which limits the drawing capability, is a potential reason for the decrease in tenacity from 10.9 cN/tex in the neat PP to 5.0 cN/tex in the PP/PP-g-MA sample and the respective decrease in modulus from 200.7 to 153.4 cN/tex that have been observed [20].

In this study, talc, which is the most common filler, was used as a conventional/standard approach for developing composite PP fibers, and those results were compared with PP fibers containing attapulgite, which presents a significant nucleating effect altering the crystallinity of the final matrix, and PP fibers containing needle-like fillers, such as wollastinite and carbon nanotubes. In addition, the effect of compatibilizer (PP-g-MA)–antioxidant interactions were studied.

## 2. Experimental

### 2.1. Materials

For the production of fibers, in all cases isotactic polypropylene was mixed with masterbatches containing the used additives, i.e., compatibilizer, antioxidant and inorganic fillers (microtalc, ultrafine talc, wollastonite, attapulgite and single-wall carbon nanotubes (SWCNT)). Table 1 summarizes important characteristics of the used materials. All of them were used as received, without any further purification. Furthermore, high-purity nanotalc (<100 nm) powder (obtained from Nanoshel LLC, Wilmington, DE, USA) was used for preparing a composite film of 90% antioxidant masterbatch and 10% talc. This sample was used for spectroscopic measurements.

### 2.2. Preparation of Composites

The prepared PP composites with microtalc, ultrafine talc, wollastonite and attapulgite contained 4 wt.% of the inorganic filler, while composites with SWCNT contained 1 wt.% of the nanofiller. All of the investigated composites contained 4 wt.% masterbatch of antioxidant, which corresponds to 0.82 wt.% of the active ingredient. In samples containing PP-g-MA as compatibilizer, 1.5 wt.% of the masterbatch (Bondyram 1001) was used. Such values for the antioxidant and compatibilizer content were chosen as typical values in common industrial practice. Moreover, the used filler content (4 wt.% for inorganic fillers and 1 wt.% for SWCNTs) represents a common composition of polymer nanocomposites, while for the development of microcomposites much higher contents are typically used (e.g., 30 wt.%). In this study, it was kept rather low, also for cost-related reasons. All the prepared samples, the used abbreviations and their composition are summarized in Table 2. Some additional information about the fillers is presented in Table S1 of the Supplementary Information file.

Firstly, the desired amounts of each masterbatch were mechanically mixed and then were inserted in a four-zone twin screw extruder (Haake Rheodrive 5001). The extrusion was performed using 25 rpm and a 3 mm die, while the temperature profile was *T*_1_ = 190 °C, *T*_2_ = 210 °C, *T*_3_ = 215 °C and *T*_4_ = 220 °C, with *T*_4_ being the temperature in the last (exit) extruder zone. The melt was cooled in a water bath that was held at 12 ± 2 °C. The produced cylindrical filament had a diameter of approximately 2 mm and was cut to pellets of 2–3 mm length. Subsequently, such pellets were inserted in a three-zone single-screw extruder (Noztek Xcalibur) in order to promote further homogenization and to produce filaments of smaller diameter (0.4–0.7 mm). The single-screw extruder was operated at 15 rpm, using a die diameter of 1.6 mm, while the temperature profile was *T*_1_ = 190 °C, *T*_2_ = 210 °C and *T*_3_ = 215 °C, with *T*_3_ being the temperature of the last (exit) zone. The melt was cooled in water bath, held at 12 ± 2 °C, and the produced filaments were collected with the aid of a filament winder (Noztek filament winder). Such filaments (of 0.4–0.7 mm diameter) were used for the subsequent solid-state drawing described in the next section.

### 2.3. Drawing of Composite Filaments

**Figure 1:**
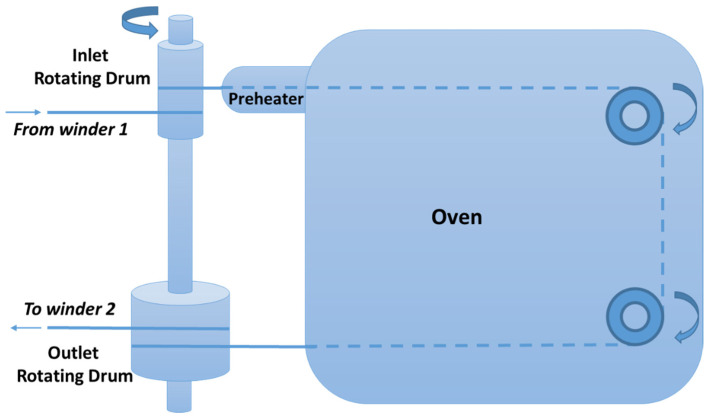
Scheme of the experimental drawing apparatus.

A scheme of the experimental drawing apparatus is presented in Figure 1. The filament from the first winder is winded twice in the inlet low-diameter drum and then it is inserted in an oven of constant temperature through a small preheating zone. The filament is then winded twice in the outlet high-diameter drum and is finally driven in the second winder. The inlet and outlet drums rotate at the same speed, of 5 rpm. In this way, the drawing ratio is equal to the ratio of their diameters.

In this study, the drawing temperature (inside the oven) was 140 ± 1 °C, while the temperature of the preheater was 120 ± 2 °C. Such temperatures are below the melting point of all the investigated composites, which, as reported in the next sections, ranged between 162 and 166 °C. Moreover, the diameter of the inlet and outlet drum was 20 and 140 mm, respectively. Consequently, the theoretical drawing ratio is equal to 7.

In order to ensure that no inhomogeneous drawing was performed, prior to the drawing, the initial filaments were marked every 50 mm, while at the end of the drawing process, only the sections of the fibers with marks at a 350 ± 10 mm distance were taken into account for further characterization. In this way, the actual obtained drawing ratio was 7.00 ± 0.2. Typically, 7–14 m of drawn fibers were produced. After drawing, the produced fibers had a diameter between 0.15 and 0.26 mm, depending on the diameter of the initial filaments.

### 2.4. Characterization

Tensile tests were performed with a Hans Schmidt & Co GmbH Universal Testing Machine ZPM equipped with a Pacific load cell (model PA6110). For each sample, 10–12 measurements were performed at room temperature using a head speed of 100 mm min^−1^. The average values and the respective mean deviation from the average value of all measurements are presented in the Results and Discussion section for each investigated composite.

The melting temperature and specific fusion enthalpy of drawn fibers were measured by differential scanning calorimetry (DSC) using a Shimadzu DSC-50 calorimeter. All measurements were carried out in nitrogen atmosphere with a gas flow rate of 20 mL min^−1^. Scans were performed in the range of 40 to 230 °C with a heating rate of 10 °C min^−1^. In all cases, the investigated DSC samples consisted of a mixture of small parts form random sections of the produced fibers.

The thermal stability of composite fibers was studied by thermogravimetric analysis (TGA) using a Shimadzu TGA-50 analyzer. Measurements were carried out in air atmosphere, in the range of 40 to 450 °C, with a heating rate of 20 °C min^−1^. A random mixture of sections of each fiber was used as sample for such TGA measurements. Moreover, constant temperature thermogravimetric analysis of the masterbatches was performed at 225 °C for 18 min. Such temperature was chosen since it is the highest temperature used in the extrusion of fibers.

Spectroscopic measurements were performed with a Biorad FTS-175 Fourier Transform Infrared Spectrometer (FTIR), in order to investigate the antioxidant–compatibilizer interactions. For this purpose, the masterbatch with the antioxidant, the masterbatch with the compatibilizer (PP-g-MA) and their blend were studied through 64 FTIR scans with a resolution of 2 cm^−1^. Their blend was prepared through mechanical mixing of the two masterbatches (one containing the antioxidant and one containing the compatibilizer) in the same proportion that was used in all fiber samples (4 parts of the antioxidant containing masterbatch and 1.5 part of the compatibilizer containing masterbatch). Subsequently, films were prepared by compression-molding the mixture at 165 °C and 200 bar for 2–3 min. Using a similar procedure, a composite containing 90% antioxidant masterbatch and 10% talc was prepared. In both cases, films of the neat masterbatches were prepared using the same procedure with the composite and blend films, in order to compare samples obtained with the same thermal process.

The morphology of some representative samples was examined with a Leica stereomicroscope (model MZ125) equipped with a Leica light source (model CLS100).

## 3. Results and Discussion

### 3.1. Constant Temperature TGA Measurements

**Figure 2:**
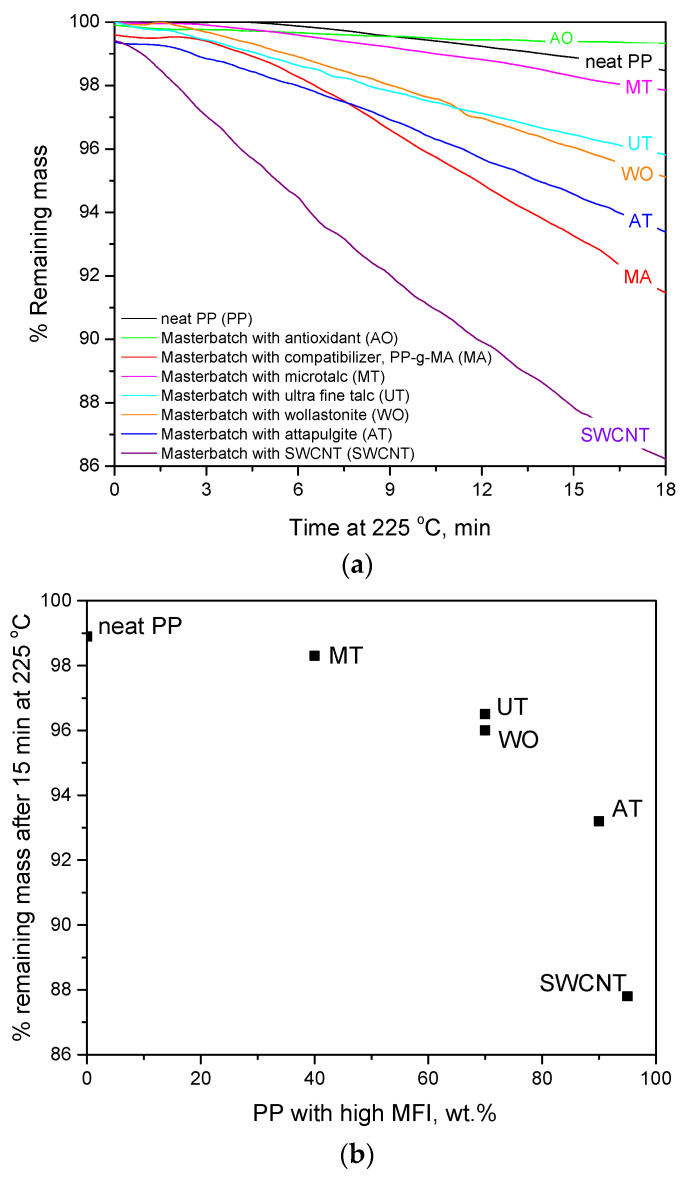
(**a**) TGA curves at constant temperature of 225 °C for the masterbatches used in this study. (**b**) Remaining mass of the masterbatches after 15 min at 225 °C as a function of their content in high MFI PP.

In order to evaluate the potential decomposition of the used raw materials during the thermal treatment and to facilitate the discussion presented in the next sections, isothermal TGA measurements were performed. In Figure 2, the TGA curves of all the used masterbatches are presented. They were obtained by measuring the mass loss at a constant temperature of 225 °C, which is the maximum temperature used in the extrusion process, for 18 min, which is far above the total residence time of a polymer pellet in the two extruders (estimated around 2 min by inserting a green pellet and measuring the time needed for the melt exiting the extruder to obtain a greenish hue).

As can be seen in Figure 2a, the neat PP is stable at 225 °C for about 5 min and appears to be the second more stable masterbatch after the masterbatch with the antioxidant. The masterbacthes with the inorganic fillers are less stable than that of pure PP. This can be attributed to two factors: (a) the fact that all masterbatches with inorganic fillers have been subjected to thermal treatment (during their preparation) and, thus, they are more thermally stressed and they decompose faster, and (b) the lower molecular weight (higher MFI, see Table 1) of the PP used for the preparation of the filler containing masterbatches.

More precisely, as can be seen in Figure 2b, a trend can be detected between the thermal stability of the masterbatches and their content in PP with higher MFI (lower molecular weight), which was subjected to thermal treatment during the preparation of masterbatches. In more detail, the neat PP masterbatch (ECOLEN HZ42Q, *MFI* = 18 g/10 min), which was not at all thermally stressed before the final mixing and spinning, contained 0 wt.% of the above-mentioned PP (*MFI* = 25 g/10 min). The microtalc masterbatch (filler content 60 wt.%) contained 40 wt.% of the high MFI and thermally stressed PP and it is the second most stable masterbatch. The masterbatches with wollastonite and ultra-fine talc contained 70% of the high MFI and thermally stressed PP and appear to have similar thermal stability and lower than the corresponding one of the microtalc masterbatch. The attapulgite masterbatch contained 90 wt.% of the high MFI and thermally stressed PP and appears to be even less stable than the above-mentioned masterbatches. Finally, the SWCNT masterbatch presented filler content equal to 5 wt.% and, consequently, contained 95 wt.% of the high MFI and thermally stressed PP. However, in the case of SWCNT, contrary to other masterbatches, in order to achieve good mixing in the preparation of the masterbatch, two extrusions were needed (i.e., one to prepare a masterbatch with 30 wt.% SWCNT content and a second extrusion/melt mixing to reach the desired, 5 wt.%, content. Thus, the SWCNT masterbatch, which was subjected to two extrusions, appears to be the least thermally stable among all masterbatches.

Thus, among the five masterbatches with inorganic fillers, the one with microtalc is much more thermally stable, since it presents the higher filler content (60 wt.% compared to 30 wt.% of the masterbatches with ultrafine talc and wollastonite, 10% wt. of the masterbatch with attapulgite and 5 wt.% of the masterbatch with SWCNT). Moreover, as shown in Figure 2a, in the initial stages (first 12 min), the masterbatch with wollastonite is slightly more stable than the one with ultrafine talc.

Undoubtedly, the masterbatch of compatibilizer presents a significant mass loss even for low heating times. Until it reaches 225 °C, it has already lost 0.5 wt.% of its mass and the mass loss rate is much higher than all other masterbatches (except the one with SWCNT). Nevertheless, extensive mass loss is initiated after approximately 2.5 min at 225 °C and, thus, degradation to only a small extent is expected during extrusions. Such results will be further discussed in the next sections.

### 3.2. Compatibilizer–Antioxidant Interactions

Talc is a phyllosilicate mineral and, similar to montmorillonite, it is used for preparing polymer nanocomposites [32]. However, the use of compatibilizer is essential for improving the distribution of the hydrophilic clay in the hydrophobic polymer matrix. Furthermore, in order to hinder the decomposition of the matrix at the high temperatures used in the extrusion process, the use of antioxidant compounds is necessary. In order to reveal the effect of fillers in various composites, firstly, one has to study the effect of such additives, i.e., compatibilizer and antioxidant.

As presented in Section 3.1, the masterbatch of compatibilizer is characterized by low thermal stability, while, as shown in Table 1, it presents much higher MFI, indicating lower polymer molecular weight. Such properties of the used compatibilizer, independently of the addition of the inorganic fillers, might have a strong influence on PP fibers. In Table 3, the results from tensile tests and TGA analysis for PP-AO drawn fibers with and without compatibilizer are presented. The tensile test results include elastic modulus, stress at break and % elongation at break. The TGA results include the onset temperature and the temperature of maximum mass loss rate. The former, *T*_97%_, was calculated as the temperature at which 3 wt.% mass loss has occurred (which corresponds to 97 wt.% remaining mass), while the latter one (*T*_max_) was calculated from the maximum at the first derivative of the TGA curve.

As can be seen in Table 3, the addition of compatibilizer (PP-g-MA) seems to improve the mechanical properties (e.g., increase in stress at break likely occurs, despite the broad distribution of measured values indicated by the average deviation from the mean), and also a similar improvement is observed in the thermal stability (increased *T*_97%_ and *T*_max_). Such results are rather unexpected, since the lower molecular weight of the PP-g-MA is expected to have a negative impact on the drawing capacity of PP and, subsequently, on the strength of fibers, which, indeed, has been observed in literature [20]. Furthermore, the compatibilizer masterbatch (see Figure 2) is much less thermally stable and, thus, a decrease in thermal stability would be expected in the PP-AO-MA sample.

A possible explanation for the increased stress at break and improvement of thermal properties might be the strong interactions between the compatibilizer (PP-g-MA) and the antioxidant, which result in better distribution of the antioxidant upon the addition of the compatibilizer. Strong polar intermolecular interactions between these two components are likely to occur since the antioxidant is of phenolic type (polar substance), while maleic anhydrate is also highly polar.

**Figure 3:**
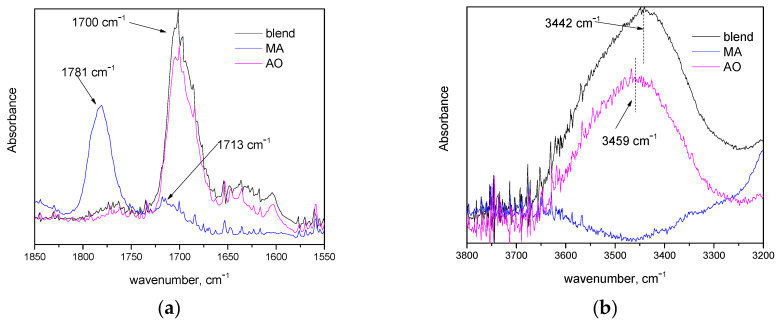
FTIR spectra of masterbatches that contain the compatibilizer (MA), the antioxidant (AO) and their blend (**a**) in the region 1550–1850 cm^−1^ and (**b**) in the region 3200–3800 cm^−1^.

In order to confirm this hypothesis, the antioxidant–compatibilizer intermolecular interactions were studied by FTIR. The obtained spectroscopic data for the masterbatches of the compatibilizer, the antioxidant and their blend are presented in Figure 3. The blend was prepared in a proportion identical to the one used for the preparation of the drawn fibers (4 parts of the antioxidant masterbatch and 1.5 part of the compatibilizer masterbatch).

In Figure 3a, the FTIR spectra, in the region 1550–1850 cm^−1^, of the compatibilizer masterbatch and the blend are presented. In this region, various >C=O vibrations occur. The absorptions at 1780 and 1713 cm^−1^ in the spectrum of compatibilizer are attributed to the symmetric >C=O stretching in anhydride groups and carboxylic acids, respectively [33,34]. Similar absorptions for PP-g-MA have been reported in literature, while the acid-related >C=O vibration was attributed to acid formation due to the anhydride hydrolysis, during the high-temperature mixing, which was catalyzed by glass fibers [34]. In the antioxidant masterbatch, the absorption of the >C=O group appears at 1700 cm^−1^, which is characteristic of conjugated carbonyl groups. In the spectrum of the blend, no contribution of the peak at 1780 cm^−1^ of PPg-MA can be detected. These observations suggest that in the blend, the absorption band of the carbonyl groups of MA has shifted (due to physical molecular interactions). In Figure 3b, the FTIR spectra in the region 3200–3800 cm^−1^ are presented. In this region, phenolic compounds exhibit absorption due to –OH groups [35]. As can be seen in Figure 3b, for the PP-g-MA, no clear peak can be detected due to the low content in –OH groups. The absorption band of -OH groups is shifted from 3459 cm^−1^ (see the spectrum of the antioxidant’s masterbatch) to 3442 cm^−1^ (see the spectrum of the blend). This suggests that –OH groups are involved in new/different hydrogen bonds in the blend. A schematic representation of the antioxidant’s better dispersion in the presence of PP-g-MA is presented in Figure S1 of Supplementary Information file, i.e., in the presence of PP-g-MA, the antioxidant molecules do not tend to form aggregates/clusters.

Based on the above, it seems that non-negligible interactions between PP-g-MA and PP-AO occur through the hydrogen bonding of the –OH of the phenolic antioxidant and the carbonyl group of MA. Such interactions enhance the dispersion of antioxidant inside the polymer matrix and less thermal degradation occurs during extrusion. Consequently, the deterioration of mechanical properties, due to thermal degradation, occurs to a lesser extent, and thus the PP-AO-MA sample appears to be superior in tensile strength.

In summary, the use of compatibilizer, although tending to decrease the mechanical strength of the fibers due to the lower molecular weight (as has been observed in the literature [20]), indirectly contributes to increased mechanical strength by promoting the thermal protection provided by the used antioxidant, which in turn results in less deterioration of the PP’s properties during extrusion.

### 3.3. Particle Nature Effect on Mechanical and Thermal Properties of PP Composite Drawn Fibers

#### 3.3.1. Crystallinity of PP Composite Drawn Fibers

In Table 4, the melting point (*T_m_*), the enthalpy of fusion (*ΔH_fus_*) and the degree of crystallinity (*X_c_*), as obtained by DSC analysis, are presented for all the drawn fiber samples. The latter property, *X_c_*, was estimated assuming that the enthalpy of fusion of 100% crystalline PP is 207 J/g [36].

All fibers present similar melting points and only minor differences are observed. Regarding the results for the enthalpy of fusion and the associated degree of crystallinity, it can be seen (Table 4) that the composites present higher crystallinity than neat PP (with or without compatibilizer). Such a property is strongly related to the to the drawing process, which, however, was identical for all samples. Consequently, the observed differences in crystallinity are attributed to the enhancement of crystallization due to heterogeneous nucleation on the polymer–filler interphase. The ability of fillers to promote heterogeneous nucleation depend both on the properties and the magnitude of the polymer–filler interphase. Thus, heterogeneous nucleation is enhanced in systems that present better distribution of filler particles, since, in this way, more available nucleation sites are present in the system.

In line with the last statement, in this study, the highest degree of crystallinity was observed for the samples containing SWCNT and attapulgite. Both SWCNT [37] and attapulgite [25] nanoparticles promote the heterogeneous nucleation of PP. Finally, as observed in Table 4, talc and wollastonite increase the crystallinity of the PP matrix to a lesser extent compared to attapulgite and SWCNTs. Talc has been reported to present some nucleation ability for PP [38], while wollastonite particles needed modification, e.g., with pimelic acid, in order to present significant nucleation ability for β-PP crystals [22].

#### 3.3.2. Thermal Stability of PP Composite Drawn Fibers

**Figure 4:**
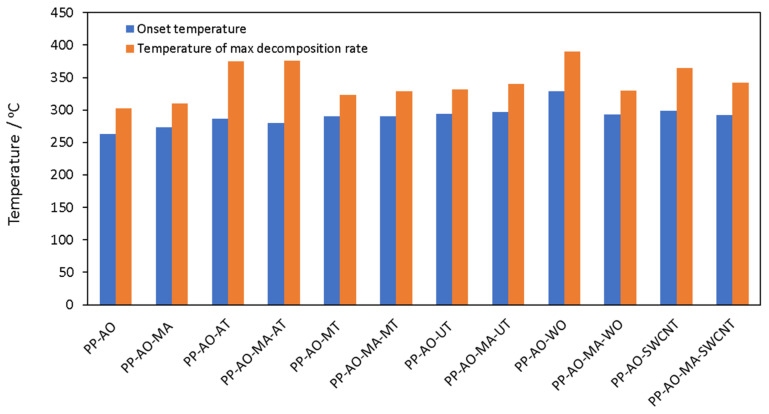
Onset decomposition temperature, *T*_97%_, and temperature at the maximum decomposition rate, *T_max_*, for all investigated drawn fibers.

The thermal stability of all drawn fibers was tested using thermogravimetric analysis. The results, considering the onset temperature, which is expressed as the temperature that corresponds to 97 wt.% remaining mass, *T*_97%_, and the temperature at maximum decomposition rate, *T_max_*, are presented in Table 5 and illustrated in Figure 4.

From the comparison of both *T*_97%_ and *T_max_* for various samples, it is revealed that all composites are more thermally stable than PP (with or without compatibilizer), which is a typical behavior of polymer composites with inorganic fillers [14]. Among all composites, the samples with attapulgite exhibited the lowest thermal stability in terms of initiation of decomposition (lowest *T*_97%_), but, at the same time, they degrade slower and exhibit one of the highest temperatures of maximum mass loss rate. Such behavior may be attributed to a relatively fast production of char, which then acts as a barrier and slows down further decomposition [14].

Furthermore, the addition of compatibilizer in the composite fibers with microtalc and ultrafine talc, only slightly affects the onset temperature, *T*_97%_, but increases (around 6 to 8 °C) the temperature of maximum mass loss rate, *T_max_*. Having in mind the discussion presented in Section 3.2, such rather small improvement of thermal stability in talc composites that contain PP-g-MA cannot be fully attributed to the better distribution of the filler, but should be also explained through the more pronounced antioxidant’s effect. Moreover, the addition of ultrafine talc results in increased thermal stability compared to that of microtalc. This is expected due to the smaller particle size of ultrafine talc that provides an increased contact surface between talc and PP.

The high polymer–filler contact surface seems to be the most important aspect also in composites with needle-like particles with a high aspect ratio, such as SCWNT and wollastonite. In more detail, the addition of only 1 wt.% SWCNT (sample PP-AO-SWCNT), which are characterized by the least hydrophilic nature among all the studied inorganic additives, significantly increases the thermal stability, i.e., increases the onset temperature, *T*_97%,_ of PP fibers around 36 °C and the temperature of maximum mass loss rate, *T_max_*, around 62 °C. Moreover, the composite with needle-like wollastonite particles, without the use of compatibilizer, exhibits the highest thermal stability from all other samples. The addition of 4 wt.% of wollastonite particles increases the onset temperature, *T*_97%_, of PP fibers around 66 °C and the temperature of maximum mass loss rate, *T_max_*, around 87 °C. Such needle-like (high aspect ratio for the case of wollastonite and very low particle size for the case of SWCNT) particles are expected to result in higher polymer–filler contact area. The dispersed particles hinder oxygen diffusion into the polymer matrix and also the diffusion of decomposition products from the polymer matrix [14].

The *T_max_* values of this study are in the same range and slightly higher than respective literature values. For example, for PP-fumed silica drawn fibers, values from 300 °C for neat PP up to 330 °C for the composite fibers with 1% silica [39] were reported. For organically modified montmorillonite, values of 348 to 387 °C for *T_max_* were reported [40]. The onset temperature for PP that was observed in this study is slightly lower than literature values [41]. Such observation can be attributed to the two extrusions that were used in this study and to the more pronounced thermal fatigue of all PP samples.

For most composites, the addition of compatibilizer, has a similar effect on the thermal stability as in the case of neat PP, that is, a minor effect on the onset decomposition temperature and a slight increase in the temperature of maximum decomposition rate. However, for the case of needle-like particles, the addition of compatibilizer caused a decrease in the thermal stability of the composites. This will be discussed in combination with the results on mechanical properties, presented in the next section.

#### 3.3.3. Mechanical Properties of PP Composite Drawn Fibers

##### Effect of Filler

**Figure 5:**
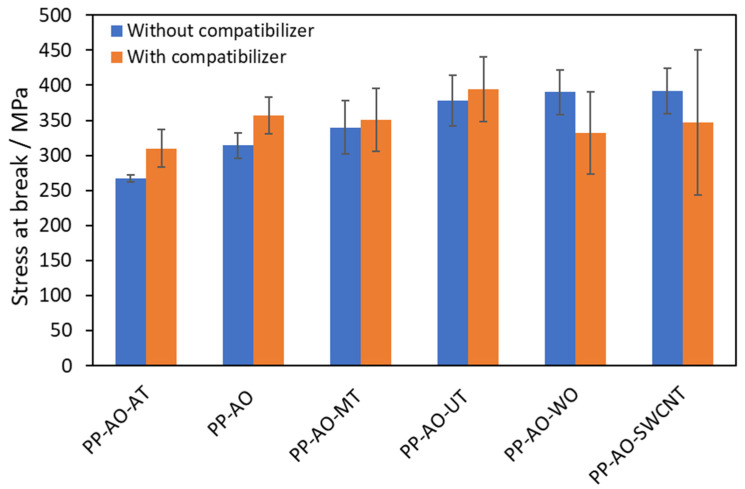
Tensile strength of the investigated drawn fibers.

The results on mechanical properties are presented in Table 5, while results for stress at break are illustrated in Figure 5. It is shown that no dramatic improvement was observed upon the addition of fillers. However, some interesting conclusions arise, despite the distribution of measured values indicated by the average deviation from the mean shown as error bars in Table 5 and Figure 5.

In more detail, in the case of attapulgite composites, the overall performance of both samples (with and without MA, see Figure 5) is rather poor, compared to that of other additives. As in the case of thermal stability, such observation suggests a poor interaction and dispersion of attapulgite particles in the polymer matrix. In the Supplementary Information file, stereoscope images (Figure S2) of the PP-AO-MA-AT sample, before and after drawing, are presented. It is observed that the presence of agglomerates is confirmed. In addition, such observation (poor performance of the attapulgite samples) may be partially attributed to the increased nucleation ability of attapulgite [25], discussed in Section 3.3.1, that leads to increased crystallinity prior to drawing, which is known to be undesirable in the drawing process [42]. Moreover, the masterbatch with the attapulgite, as already discussed in Section 3.1, exhibited the second poorest thermal stability, partially due to the presence of PP with higher MFI than the one of the neat PP masterbatch. The higher MFI (lower molecular weight) is likely to hinder the drawing process.

The addition of microtalc, without any compatibilizer (PP-AO-MT composite) seems to increase the elastic modulus and tensile strength of the respective PP-AO sample. On the contrary, the addition of ultrafine talc seems to have a more considerable effect on tensile strength than the addition of microtalc. It is well known that the particle size affects the properties of the composites and that the smaller the size of the distributed filler particles, the more pronounced the improvement, since the filler–polymer contact area becomes more considerable. However, despite the fact that without compatibilizer the improvement of mechanical properties is rather low, even this low improvement is unexpected. This will be further discussed in Section 3.4.

The use of SWCNT, even at the low content of 1% wt., results in a considerable increase in tensile strength (sample PP-AO-SWCNT). As discussed in the introduction section, without appropriate modification of SWCNT, the respective PP composites exhibit similar (or inferior in some cases) properties to neat PP and only the modification of fluorinated CNT resulted in a considerable increase in tensile strength, due to covalent bonding between PP and -F of the fluorinated CNT [31]. The difference of the melt flow index of the various PP and other masterbatches, used in this study and in the literature, along with the different drawing ratio and thermal processing do not allow for strict comparisons. In the literature, it was reported that the tensile strength of PP fibers containing 1% CNTs and drawn up to a ratio of 7.9 was 479 MPa, while the respective value for the sample without carbon nanofibers was 471 MPa [10]. Such values are close to the one observed in this study (392 MPa, using a draw ratio of 7.0 and 1% SWCNT).

Equally good results, with the case of SWCNT, in terms of tensile strength, were observed for PP–wollastonite composite (PP-AO-WO). Without the use of compatibilizer, this sample presents similar tensile strength with the ultrafine talc sample that contains compatibilizer (PP-AO-MA-UT) and with the SWCNT-containing sample (PP-AO-SWCNT). As in the case of thermal stability, the tensile strength of such two composites containing needle-like particles is the highest observed. It is worth mentioning that in the case of PP-AO-WO samples it was very difficult to observe agglomerates with the stereomicroscope, both in drawn and non-drawn samples, as shown in Figure S3 of the Supplementary Information file.

##### Effect of Compatibilizer

The addition of compatibilizer (PP-g-MA) in PP-AO and in all the composites, except the SWCNT and wollastonite composites, seems to cause a small improvement in the tensile strength (as in the case of thermal stability that was discussed in a previous section). This improvement can be mainly attributed to the compatibilizer–antioxidant interactions discussed in Section 3.2, rather than to the filler–compatibilizer interactions. On the contrary, in the case of needle-like particles (SWCNT and wollastonite), a decrease in the tensile strength is observed with the addition of compatibilizer. Moreover, a rather peculiar effect of compatibilizer can be recognized, since the addition of PP-g-MA seems to result in less homogeneous materials. This can be easily detected from Figure 5, where it can be seen that in all samples containing PP-g-MA, the diversity of the obtained values (indicated by the reported error bars) is greater than in the respective sample without compatibilizer. This can be attributed to competitive phenomena of the interactions between compatibilizer and antioxidant, between compatibilizer and filler, and the interactions of each additive with PP, which, as a consequence, result in the formation of regions of different composition, e.g., aggregates of particles. By taking into account (a) the positive effect on the mechanical properties, due to the enhanced dispersion of antioxidant when PP-g-MA is added (see Section 3.2), and (b) the positive effect caused by the addition of the filler particles, it can be concluded that the addition of PP-g-MA, due to the above-mentioned competitive effects, may overall interfere (SWCNT and wollastonite) or enhance the influence of particles (micrortalc and ultra-fine talc). For example, PP-g-MA was used in the SWCNT composites only to induce the dispersion of antioxidant, according to the observations of Section 3.2. Since it is a hydrophilic compatibilizer, it is not expected to promote the distribution of SWCNTs in the PP matrix. Indeed, the addition of such an additive hindered the uniform dispersion of SWCNTs, as indicated by the diversity of the obtained values. Thus, upon the addition of PP-g-MA, the hindering of SWCNT dispersion prevailed over the better antioxidant dispersion.

Notwithstanding the above-mentioned explanation, the dispersion of SWCNT may be hindered by the addition of the low molecular weight compatibilizer for an additional reason related to the needle-like geometry of such particles. More specifically, among other methods [43], uniaxial mechanical stretching (at 100 °C) has been reported to align carbon nanotubes inside a polymer matrix and that this alignment is retained at room temperature [30]. Thus, the presence of short chains (PP-g-MA), which cannot be drawn to the same extent as the high molecular weight chains, may also interfere with the alignment of SWCNT. An analogous explanation can be given for the case of the wollastonite. As mentioned already, the two samples with needle-like particles (SWCNT and wollastonite) and without compatibilizer yielded the best results among all samples, regarding both thermal stability and mechanical strength. Compared to PP-AO and PP-AO-MA fibers, an improvement was observed in the sample with wollastonite (PP-AO-WO). Since no compatibilizer was used, such improvement is attributed to the interaction of PP with the wollastonite needle-like particles. Due to the non-polar nature of PP, a strong interaction with wollastonite is not expected. In addition, wollastonite is widely used as filler in typical PP and other polymer microcomposites (not especially for drawn fibers). In such applications, high loadings, e.g., 30 wt.%, are required in order to observe considerable improvement of composite properties [24]. Thus, the improvement of mechanical and thermal properties that were observed in this study should be attributed mainly to the specific geometry of such filler particles, which present a needle-like structure with a high aspect ratio. It is reasonable to assume that, similar to SWCNTs [30], during drawing, their geometry favors their alignment and hinders their coalescence and thus a more uniform dispersion is accomplished. In addition, such alignment causes a more symmetrical stress distribution compared to that of randomly dispersed particles (e.g., talc), when tensile forces are applied to the fiber and, thus, the load is shared more uniformly in the crystalline and amorphous regions of PP. In such terms, the deterioration of the properties of the PP–wollastonite and PP–SWCNT composites with the addition of PP-g-MA might be explained. The effect of the lower molecular weight of PP-g-MA, most probably, prevails and disturbs the wollastonite distribution and alignment along the PP chains.

### 3.4. Antioxidant as Compatibilizing Agent

As it was discussed in Section 3.2, interactions between compatibilizer and antioxidant occur. In Section 3.3, it was discussed that the use of compatibilizer in some cases, e.g., talc, improves mechanical properties, while in other cases it causes their deterioration. All these indicate that in composites with a significant number of additives (antioxidant, compatibilizer, and inorganic fillers), various interactions, including synergistic or competitive effects, take place. In Section 3.3.3, in the discussion of the influence of talc on the mechanical properties of PP, it was mentioned that the improvement of the mechanical properties by the use of talc without any compatibilizer is rather unexpected. This creates a suspicion that the antioxidant may act as a compatibilizer between, at least, talc and PP. This would not be surprising since antioxidants of phenolic type, besides polar groups (hydroxyl groups), contain non-polar chains and have a similar structure to that of surfactants.

**Figure 6:**
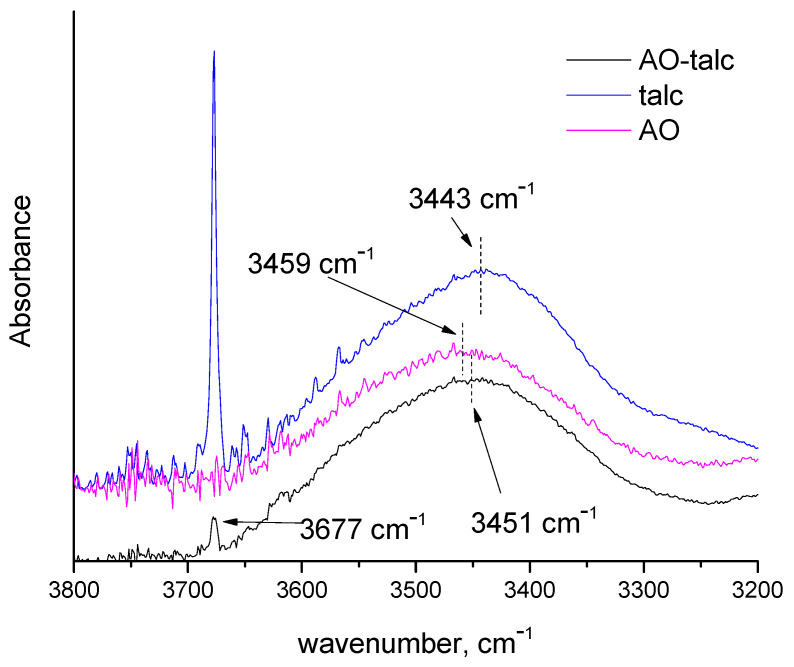
FTIR spectra of talc, of the masterbatch of the antioxidant (AO) and their composite material (AO-talc) in the region 3200–3800 cm^−1^.

In order to test such a hypothesis, a talc sample and a composite film of antioxidant masterbatch and talc were studied with FTIR (Figure 6). As can be seen, the absorption band of antioxidant hydroxyls is slightly shifted in the composite material, suggesting different hydrogen bonding behavior. In the spectrum of talc, the sharp peak at 3677 cm^−1^ is assigned to Mg_3_OH vibrations and the broad peak at around 3443 cm^−1^ is assigned to hydroxyl groups [44] related to absorbed water [45]. In the spectrum of the composite material, the sharp peak of talc is low and, consequently, the peak related to water should be accordingly low. Thus, the observed shift of the antioxidant hydroxyls’ peak in the spectrum of the composite cannot be solely attributed to the water of talc. However, the water adsorbed in talc may play a significant role in the talc–antioxidant interactions. The water adsorbed, in talc, may interact with hydroxyl groups of the phenolic antioxidant through hydrogen bonding and leave the non-polar region of the antioxidant readily available to PP. The important role of water in the exfoliation of montmorillonite by hydrophilic compounds such as starch has been reported [46].

## 4. Conclusions

Various PP composite drawn fibers were prepared by melt extrusion and high-temperature solid-state drawing at a draw ratio of 7. Antioxidant addition during extrusion is a common industrial practice. It was observed that the PP-g-MA, which was initially selected for inducing filler–PP compatibility, interacts strongly with the phenolic-type antioxidant and, thus, enhances thermal protection during extrusion, which in turn results in less deterioration of mechanical properties. Consequently, the use of such compatibilizer, independently of the use of inorganic fillers, yields PP fibers with more attractive properties.

Attapulgite exhibited the poorest results, most likely related to poor interactions with PP and to its increased nucleation ability that induces polymer crystallinity prior to the drawing process. Regarding the talc composites, the use of talc with a smaller particle size gave better results in terms of mechanical properties and thermal stability. Small differences in such composites, with or without compatibilizer, are attributed to the compatibilizer–antioxidant interactions. An unexpected slight improvement of the mechanical properties in the case of PP–talc composite fibers without using compatibilizer seems to arise from the compatibilizing effect of the antioxidant. The fillers with needle-like morphology, without the use of PP-g-MA as compatibilizer, yielded the best results. The improvement of PP properties with the addition of wollastonite and SWCNT is likely related to their geometry, since it has been reported that during drawing the needle-like particles with a high aspect ratio align along the axis of PP chain alignment [30]. In both samples, the addition of PP-g-MA causes a deterioration of thermal stability and mechanical strength.

It is suggested that the compatibilizer’s content and other parameters, such as the antioxidant’s and filler’s content and the drawing ratio, should be optimized in order to achieve maximum tensile strength and thermal stability.

## Figures and Tables

**Table 1:** Important characteristics of the used materials.

Material	Abbreviation	Characteristics ^1,2,3,4^	Supplier
Isotactic PP (ECOLEN HZ42Q)	PP	*MFI* = 18 g/10 min, *TS* = 33 MPa, *T_m_* = 168–171 °C	Hellenic Petroleum S.A., Thessaloniki, Greece
Masterbatch with compatibilizer (Bondyram 1001)	MA	PP grafted with maleic anhydride (PP-g-MA). MA content 1%, *MFI* = 100 g/10 min, *T_m_* = 160 °C	Polyram Plastic Industries LTD, Gilboa, Israel
Masterbatch with antioxidant (KRITILEN^®^ AO PP9216)	AO	PP with 20.5 wt.% antioxidant (combination of phosphite and phenolic types)	Plastika Kritis S.A., Heraklion, Greece
Masterbatch with microtalc (KRITILEN^®^ D05-00014)	MT	PP with 60 wt.% microtalc (*D*_50_ = 1.7 μm) *MFI* ^5^ = 25 g/10 min	Plastika Kritis S.A., Heraklion, Greece
Masterbatch with ultra-fine talc (KRITILEN^®^ D05-00046)	UT	PP with 30 wt.% ultra-fine (*D*_50_ = 0.7 μm) *MFI* ^5^ = 25 g/10 min	Plastika Kritis S.A., Heraklion, Greece
Masterbatch with wollastonite (KRITILEN^®^ D05-00047)	WO	PP with 30 wt.% wollastonite of high aspect ratio (*D*_50_ = 3 μm) *MFI* ^5^ = 25 g/10 min	Plastika Kritis S.A., Heraklion, Greece
Masterbatch with attapulgite (KRITILEN^®^ D05-00048)	AT	PP with 10 wt.% attapulgite *MFI* ^5^ = 25 g/10 min	Plastika Kritis S.A., Heraklion, Greece
Masterbatch with SWCNT (KRITILEN^®^ D05-00067)	SWCNT	PP with 5 wt.% SWCNT *MFI* ^5^ = 25 g/10 min	Plastika Kritis S.A., Heraklion, Greece

^1^*MFI*: melt flow index, ^2^*TS*: tensile strength, ^3^*T_m_*: melting point, ^4^*D*_50_: mass-median-diameter, ^5^ MFI of the PP used for the preparation of the masterbatch and not the MFI of masterbatch itself.

**Table 2:** Prepared composites and their composition.

Composite	Filler Type	Filler Content (wt.%)	Antioxidant (wt.%)	Copatibilizer Content ^1^ (wt.%)
PP-AO	-	-	0.82	-
PP-AO-MA	-	-	0.82	1.5
PP-AO-MT	Microtalc	4	0.82	-
PP-AO-MA-MT	Microtalc	4	0.82	1.5
PP-AO-UT	Ultrafine talc	4	0.82	-
PP-AO-MA-UT	Ultrafine talc	4	0.82	1.5
PP-AO-WO	Wolllastonite	4	0.82	-
PP-AO-MA-WO	Wolllastonite	4	0.82	1.5
PP-AO-AT	Attapulgite	4	0.82	-
PP-AO-MA-AT	Attapulgite	4	0.82	1.5
PP-AO-SWCNT	Single-Wall Carbon Nanotubes	1	0.82	-
PP-AO-MA- SWCNT	Single-Wall Carbon Nanotubes	1	0.82	1.5

^1^ On masterbatch base.

**Table 3:** Results from tensile tests and TGA for PP-AO drawn fibers with and without compatibilizer.

Sample	Elastic Modulus/MPa	Stress at Break/MPa	% Εlongation at Break	^1^*T*_97%_/°C	^2^*T_max_*/°C
PP-AO	2035 ± 152	314 ± 18	165 ± 13	263	303
PP-AO-MA	2458 ± 235	357 ± 26	168 ± 12	273	310

^1^*T*_97%_: onset temperature, ^2^*T_max_*: temperature at the maximum mass loss rate.

**Table 4:** Results from DSC measurements.

Sample	*T_m_*/°C	Δ*H_fus_*/J g^−1^	% *X_c_*
PP-AO	164	83.1	40
PP-AO-MA	162	83.6	40
PP-AO-MT	165	89.0	43
PP-AO-MA-MT	165	86.7	42
PP-AO-UT	162	88.9	43
PP-AO-MA-UT	164	91.2	44
PP-AO-WO	165	91.8	44
PP-AO-MA-WO	167	89.0	43
PP-AO-AT	165	103.2	50
PP-AO-MA-AT	166	99.4	48
PP-AO-SWCNT	165	102.7	50
PP-AO-ΜA-SWCNT	164	96.6	47

*T_m_*: melting temperature, Δ*H_fus_*: enthalpy of fusion, *X_c_*: degree of crystallinity.

**Table 5:** Tensile test and TGA results for all investigated samples.

Sample	Elastic Modulus, MPa	Stress at Break, MPa	% Elongation at Break	*T_97%_*, °C	*T_max_*, °C
PP-AO	2035 ± 152	314 ± 18	165 ± 13	263	303
PP-AO-MA	2458 ± 235	357 ± 26	168 ± 12	273	310
PP-AO-MT	2183 ± 230	340 ± 38	159 ± 14	290	323
PP-AO-MA-MT	2468 ± 393	351 ± 45	154 ± 12	290	329
PP-AO-UT	2680 ± 368	378 ± 36	173 ± 10	294	332
PP-AO-MA-UT	2206 ± 384	394 ± 46	176 ± 17	297	340
PP-AO-WO	2611 ± 305	390 ± 32	178 ± 40	329	390
PP-AO-MA-WO	2525 ± 585	366 ± 59	165 ± 10	293	330
PP-AO-AT	2016 ± 324	267 ± 05	184 ± 11	287	375
PP-AO-MA-AT	2332 ± 150	310 ± 27	181 ± 10	280	376
PP-AO-SWCNT	2264 ± 283	392 ± 32	187 ± 21	299	365
PP-AO-MA-SWCNT	2719 ± 656	347 ± 103	170 ± 06	292	342

## Data Availability

Not applicable.

## Supplementary Materials

The following supporting information can be downloaded at: https://www.mdpi.com/article/10.3390/polym14020260/s1, Figure S1: Schematic representation of the better antioxidant dispersion in presence of the compatibilizer; Figure S2: Stereoscope images of the PP-AO-MA-AT sample: (**a**) before drawing and (**b**) after drawing. The arrows indicate the presence of agglomerates; Figure S3: Stereoscope images of non-drawn PP-AO-WO samples: (**a**) Image showing a representative uniform fiber area and (**b**) Image showing the presence of agglomerate (very rare and not representative for this composite); Table S1: Information for the fillers that were used for the preparation of masterbatches.
