# Supplementary material for: Experimental Investigation of Polypropylene Composite Drawn Fibers with Talc, Wollastonite, Attapulgite and Single-Wall Carbon Nanotubes
Source: Polymers (Basel). 2022 Jan 9;14(2):260. doi: 10.3390/polym14020260 (PMC8777760; doi:10.3390/polym14020260)
Supplement: Supplementary file 1 [file polymers-14-00260-s001.zip › polymers-1499639-supplementary.pdf]

polymers

Supplementary Information for

Experimental Investigation of Polypropylene Composite Drawn Fibers with Talc, Wollastonite, Attapulgite and Single Wall Carbon Nanotubes

Costas Tsioptsias[1], Konstantinos Leontiadis[1], Stavros Messaritakis[2], Aikaterini Terzaki[2], Panagiotis Xidas[3], Kyriakos Mystikos[3], Evangelos Tzimpilis[1] and Ioannis Tsivintzelis[1]*

[1]Department of Chemical Engineering, Aristotle University of Thessaloniki, University Campus, GR-54124, Thessaloniki, Greece.
[2]Plastika Kritis S.A., R Street, Industrial Area of Heraklion, GR-71408, Heraklion, Crete, Greece.
[3]Thrace Nonwovens & Geosynthetics S.A., Magiko, GR-67100, Xanthi, Greece
*Correspondence: authors, tioannis@cheng.auth.gr (I. Tsivintzelis), ktsiopts@gmail.com (Costas Tsioptsias)

In Table S1 some information about the fillers that were used for the preparation of the masterbatches, are given.

Table S1. Information for the fillers that were used for the preparation of masterbatches.

Name	Chemical formula	Type	Size
Microtalc	$Mg_3Si_4O_{10}(OH)_2$	Phyllosilicate	D_{50} = 1.7 μm
Ultrafine talc	$Mg_3Si_4O_{10}(OH)_2$	Phyllosilicate	D_{50} =0.7 μm
Attapulgite	$(Mg,Al)_2Si_4O_{10}(OH)\cdot 4(H_2O)$	Phyllosilicate	
Wollastonite	$CaSiO_3$	Silicate needle-like	D_{50} = 3μm
Single wall carbon nanotubes	C	Carbonaceous needle-like	

In Figure S1 a schematic representation of the better dispersion of the antioxidant inside the PP matrix, in presence of the compatibilizer (PP-g-MA), is given. As can be seen, in absence of PP-g-MA the molecules of the phenolic type antioxidant are expected to self-aggregate and, consequently, their dispersion is hindered. In presence of PP-g-MA, the hydrogen bonding between the -OH group of the antioxidant and the oxygens of MA group leads to a better dispersion of the antioxidant molecules into the PP matrix.

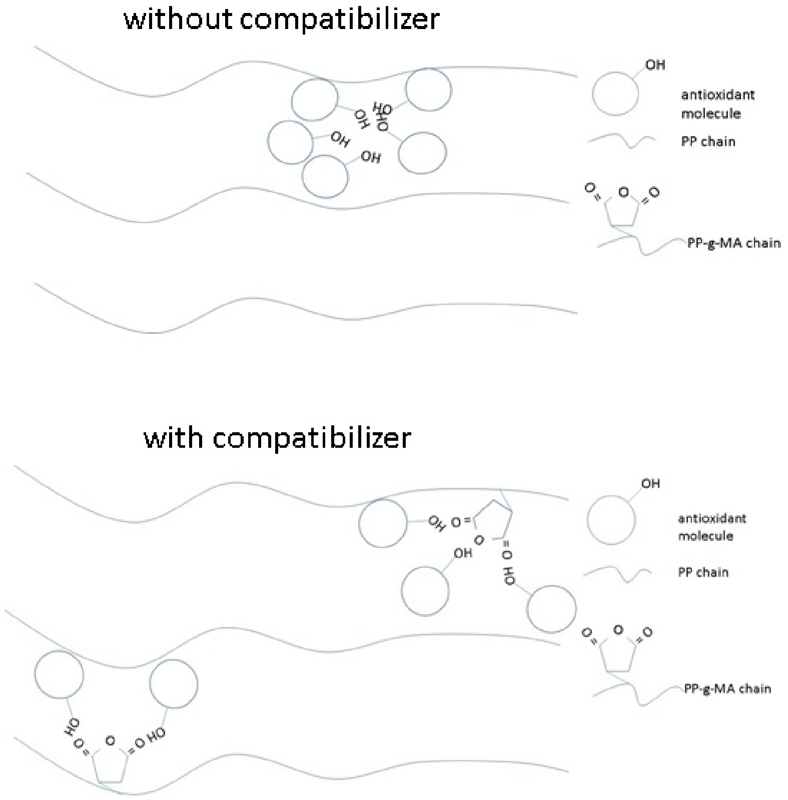

Figure S1. Schematic representation of the better antioxidant dispersion in presence of the compatibilizer.

In Figures S2 and S3, images from stereoscope are presented for the PP-AO-MA-AT and PP-AO-WO samples. In the case of the PP-AO-MA-AT sample, aggregates can be rather often detected (indicated by arrows in Figure S2). On the contrary, for the PP-AO-WO sample the observation of aggregates was extremely rare.

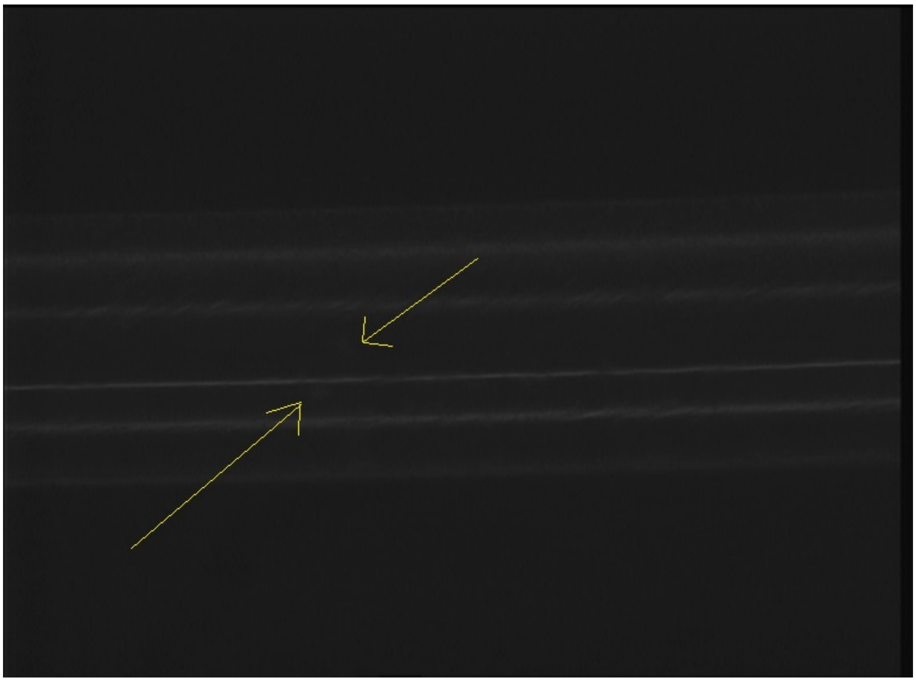

(a)

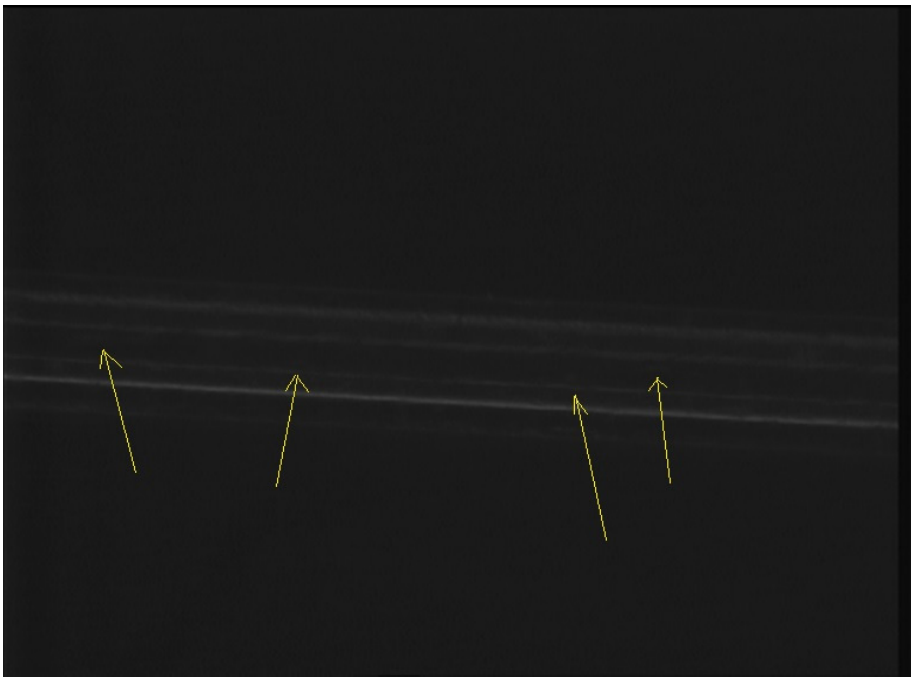

(b)

Figure S2. Stereoscope images of the PP-AO-MA-AT sample: (**a**) before drawing and (**b**) after drawing. The arrows indicate the presence of agglomerates.

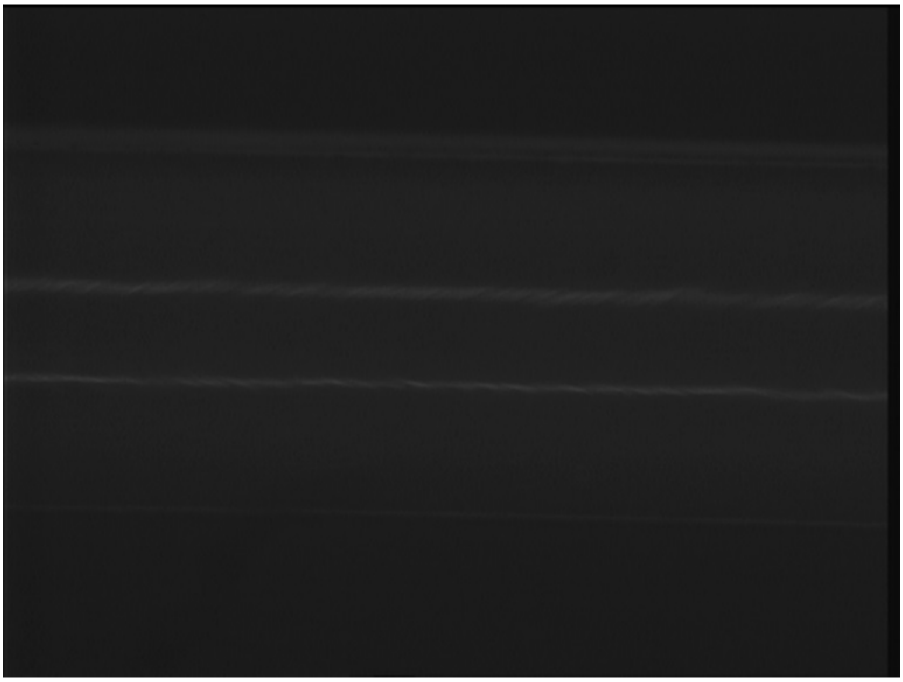

(a)

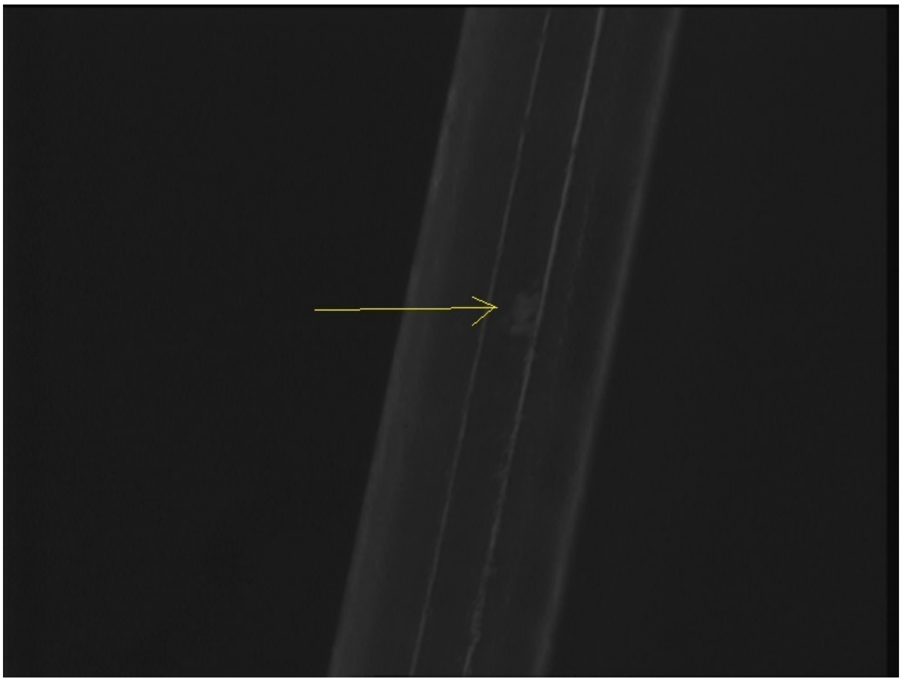

(b)

Figure S3. Stereoscope images of non-drawn PP-AO-WO samples: (**a**) Image showing a representative uniform fiber area and (**b**) Image showing the presence of agglomerate (very rare and not representative for this composite).
